# Supplementary material for: Enhancing user perception and drone flight control through AR-based object projection
Source: PLoS One. 2026 Apr 30;21(4):e0346937. doi: 10.1371/journal.pone.0346937 (PMC13132239; doi:10.1371/journal.pone.0346937)
Supplement: S1 File — This file contains additional tables supporting the results of this study. • Table A. Factor loadings for operational metrics. • Table B. Factor loadings for questionnaire items. • Table C. Effect sizes (Hedges’ g) and 95% confidence intervals. (PDF) [file pone.0346937.s001.pdf]

Supporting Information

S1 File. Supporting tables.

Table A. Factor loadings for operational metrics.
This table presents the factor loading matrix obtained from the factor analysis of controller operation data.

Item	Factor1	Factor2	Factor3
Total Number of Operations	0.991	0.121	-0.045
Number of Continuous Movements	0.950	0.214	0.091
Number of Repeated Movements	0.930	-0.233	-0.116
Operations per Unit Time	0.836	0.258	0.440
Cumulative Displacement	0.802	0.117	-0.369
Average Time Interval Between Actions	-0.748	-0.222	-0.523
Average Duration per Operation	-0.683	-0.310	-0.586
Mean Controller Operation Acceleration	0.578	-0.568	0.160
Mean Operation Size1	-0.582	0.801	-0.061
Average Maximum Control Displacement	-0.599	0.793	-0.085
Movement Smoothness	-0.407	0.791	0.233
Variation in Maximum Control Displacement	-0.277	0.752	0.359
Mean Controller Operation Speed	-0.438	0.725	-0.202
Mean Operation Size2	-0.644	0.706	-0.209
Number of Direction Reversals	0.289	0.619	0.144
Total Time	0.407	-0.291	-0.699
Average Size per Unit Duration	0.479	0.603	0.617
Number of Collisions (Wall)	0.035	0.017	-0.224
Crash	0.048	-0.023	-0.219
Number of Screen Exits	0.127	-0.074	-0.140
Number of Collisions (Pole)	0.082	0.062	0.021
Idle Time	-0.058	0.098	0.000

Table B. Factor loadings for questionnaire items.

This table shows the factor loadings derived from the questionnaire responses.

Item	Factor1	Factor2	Factor3
Likeability	-0.922	0.083	-0.063
Interaction	-0.922	-0.118	-0.084
Fun	-0.743	-0.019	0.117
Trustworthiness	-0.688	-0.139	0.057
Intelligence	-0.572	-0.486	0.175
Difficulty	-0.549	0.037	0.065
Friendliness	-0.518	0.479	-0.325
Stability	-0.417	-0.270	-0.003
Complexity	0.381	-0.159	-0.288
Age	-0.117	-0.807	0.289
Animal Likeness	-0.227	0.693	-0.249
Gender	-0.014	0.559	-0.436
Strength	-0.021	-0.430	-0.197
Size	0.126	-0.525	-0.719
Weight	0.348	-0.389	-0.442

Table C. Effect sizes (Hedges' g) and 95% confidence intervals.

Effect sizes and corresponding confidence intervals comparing Condition A and B.

Item	Hedges' g	95% CI Lower	95% CI Upper	Interpretation
Complexity	0.094	-0.69	1.07	Not significant
Difficulty	-0.075	-0.97	0.69	Not significant
Strength	-0.517	-1.49	-0.12	Small–moderate negative effect
Stability	-0.187	-1.11	0.45	Not significant
Size	-0.101	-0.76	0.47	Not significant
Weight	-0.280	-1.10	0.24	Not significant
Animal likeness	1.210	1.16	2.46	Large significant positive effect
Friendliness	0.550	0.21	1.88	Moderate significant positive effect
Intelligence	-0.633	-1.69	-0.31	Small–moderate negative effect
Trustworthiness	-0.270	-1.37	0.32	Not significant
Age	0.796	0.41	1.40	Large significant positive effect
Gender	-0.043	-0.54	0.44	Not significant
Fun	-0.072	-0.67	0.48	Not significant
Interaction	-0.073	-0.67	0.48	Not significant
Likeability	0.376	-0.06	0.82	Not significant
