# Supplementary material for: Enhancing user perception and drone flight control through AR-based object projection
Source: PLoS One. 2026 Apr 30;21(4):e0346937. doi: 10.1371/journal.pone.0346937 (PMC13132239; doi:10.1371/journal.pone.0346937)
Supplement: Attachment — Submitted filename: Response to Reviewers.docx [file pone.0346937.s002.docx]

Manuscript Number: PONE-D-25-33316

Article Title: “Enhancing user perception and drone flight control through AR-based object projection”

To: PLOSOne Editor
Re: Response to reviewers

Dear Editor,

We are grateful to the two reviewers for their thorough and precise review of our
manuscript. In response to the valuable points raised by each reviewer, we have made
revisions accordingly. We have summarized the modifications below. We hope the
revised manuscript is now suitable for your consideration.

- Regarding Editor:

Q1-1 The study includes only 21 participants, limiting generalizability. Additional details on participant demographics (age, gender balance, prior drone/AR experience) would strengthen the context for interpreting results. In order to justify the participants size, I suggest to review this article "Perceptions on safety climate: a case study in the Omani construction industry".

A1-1We appreciate the reviewer’s comment regarding the limited number of participants and the need for additional demographic information. In response, we have added details on participant demographics to the Methods section: the study included 21 participants (n=21n = 21n=21), comprising 12 males and 9 females, aged between 18 and 50 years (mean = 24.9, variance = 52.5). Seven participants had prior experience using drones, whereas 14 had no such experience.

We also note that, within the scope of our study, we have conducted an interpretation of these participant attributes to better contextualize the results.

Furthermore, to justify the sample size for an exploratory study, we have cited Umar (2016), who conducted qualitative research on safety climate in the Omani construction industry with six participants. This supports the view that small samples can provide rich and valuable insights relevant to the research questions.

We have added a sentence addressing this comment at the end of all questionnaire items, on page 2, line 2, and page 3, line 45-50, and page5-6, line117-124.

Q1-2 The discussion acknowledges the use of 2D projections, but more analysis of how 3D projections or real-world deployment might alter findings would add depth. See this recent study which consider a number of technical features "Applications of drones for safety inspection in the Gulf Cooperation Council construction".

A2-2 We appreciate the reviewer’s suggestion regarding the exploration of 3D projections and real-world deployment. This is an important direction for future research. As indicated in the study on drone applications for safety inspection in the Gulf Cooperation Council construction industry, technical features such as camera movability and perspective can significantly influence safety-related tasks. In our study, we used a 2D AR projection, which allows operators to perceive the drone’s projected guidance on a flat plane. If a 3D device such as HoloLens were used, the added depth information might enhance spatial understanding and safety awareness. On the other hand, for devices like Oculus, where the real-world scene is first converted into camera feed, the benefits of 3D projection may be limited, as the operator still perceives the scene primarily in a planar view. Future work will examine how different AR/VR devices and true 3D visualization can affect operator perception and safety performance in real-world construction environments.

We have addressed this comment by adding a sentence in Introduction (page 1) and on page 14, line 335-342.

Q1-3. The highly controlled laboratory environment (green screen, stickers) may not reflect real-world drone operation. A clearer discussion of ecological validity and potential field applications would enhance impact. Suggest to review this article for validation "Developing a safety climate assessment tool for Omani construction industry"

A1-3 We appreciate the reviewer’s insightful suggestion. In this study, we initially aimed to implement the AR projection in a real-world setting using HoloLens; however, at the time of development, the computational performance of available devices made real-time projection on a moving drone technically difficult. Therefore, we conducted the experiment in a controlled laboratory environment using a green screen and visual markers to ensure detection stability and safety.

In line with *Umar (2022)*, who developed and validated a safety climate assessment tool under controlled conditions before proposing its application in real construction projects, we believe that our laboratory-based validation serves as a necessary first step toward future field implementation. As device performance and real-time processing capabilities continue to improve, the proposed method could be extended to outdoor environments and actual drone operations.

We have added a sentence addressing this comment at the end of all questionnaire items, on page 13, line 292-303.

Q1-4 While factor analysis and t/Wilcoxon tests are described, reporting effect sizes (e.g., Cohen’s d) and confidence intervals would improve transparency and allow readers to assess practical significance. I suggest to review this document which provide a number statistical analysis which could be useful in your study "Developing toolkits and guidelines to improve safety performance in the construction industry in Oman"

A1-4 We appreciate the reviewer’s suggestion to report effect sizes and confidence intervals. Following this recommendation, and adopting the statistical approach described by Umar et al., we calculated Hedges’ *g* and the 95% confidence intervals for all questionnaire items (Table 5).The results show that items related to operational behavior (e.g., complexity, difficulty, stability, size, weight) have effect sizes near zero with CIs crossing zero, indicating that AR projection did not affect operational performance.In contrast, several impression-related items (Animal likeness, Friendliness, Age-related perceptions) have positive effect sizes with CIs above zero, suggesting that the AR projection influenced these perceptual evaluations.

We have added a sentence addressing this comment at the end of all questionnaire items, on page 8-10, line 217-232.

■Regarding Reviewer #1:

Q2-1 A large number of participants are engaged in the experiment designed. Better control and experiment design should be conducted to provide more objective comparison.

A2-1 Thank you for your valuable comment. We acknowledge that a more rigorous experimental design is an important future task.

We have added a sentence addressing this comment at the end of all questionnaire items, on page 14, line 335-336.

Q2-2. The novelty of the study should be much better highlighted in the abstract. Contributions and limitations of the research should be better discussed as well in the Conclusion.

A2-2. We thank the reviewer for this valuable comment.
In response, we have revised the abstract to more explicitly highlight the novelty of the study, emphasizing that this is the first experimental investigation of how AR-based appearance changes in drones affect user operational behavior and impressions.

We have addressed this comment by adding a sentence in the Abstract and Introduction (page 1)

Q2-3. There are 25 reference papers cited in the article which is in the low end. Among 25 reference papers, only 6 of them are published after 2020.

A2-3. We thank the reviewer for this comment. As also suggested by other reviewers, we have added five additional references([6],[27],[28],[29],[30]) published after 2020 to better reflect recent research. At the same time, we have retained the references published before 2020, because they provide essential background and foundational studies that are still relevant to the topic and help contextualize our research.

Q2-4 The accuracy of the AR projection or alignment is something of interesting that should be discussed in more detail. Also, drone flighting is a dynamic process highly related to robotic path planning. Some further elaboration is expected.

A2-4 We thank the reviewer for highlighting the importance of AR projection accuracy and the dynamic nature of drone flight control. In our experimental setup, there was an inherent **trade-off between flight safety and AR detection precision**. While a smaller drone was preferable to ensure participant safety during close-range operation, **our method relied on detecting the drone by its color, meaning that a larger drone could function as a larger marker, thereby improving AR alignment and projection accuracy**. Considering this trade-off, we selected the **DJI Mini**, which provided an optimal compromise between safety and tracking stability.

Furthermore, within the constraints of the available equipment, we made every effort to **maximize AR projection accuracy** by optimizing environmental settings, including the installation of a green screen, as well as adjusting the drone’s color to enhance its function as an AR marker.

We have added a sentence addressing this comment at the end of all questionnaire items, on page 5, line 105-112.

Q2-5 Most of the photos should be rotated by 90 degree clockwise to allow easier reading.

A2-5 Thank you for pointing this out. We have confirmed that the original image files are correctly oriented. However, when uploaded to the PLOS submission system, only the landscape-oriented images are automatically rotated by 90 degrees. Unfortunately, this rotation occurs within the submission platform and cannot be controlled by the authors.
We would like to note that the images are correctly prepared in the manuscript, and we expect this issue to be resolved during the final production stage by the editorial team. Therefore, we would like to proceed with the current submission.

Q2-6 The state-of-the-art below can help readers better understand the complexity of the research: - Xu P., et al (2025). SafeSpect: Safety-First Augmented Reality Heads-up Display for Drone Inspections. CHI 2025, Yokohama, Japan.

A2-6. We thank the reviewer for the valuable suggestion. We have added a discussion of Xu et al. (2025) in both the Introduction and Related Work sections. In the Introduction , we briefly mention SafeSpect as an example of a safety-first AR interface that improves situational awareness and reduces cognitive load for drone pilots, highlighting the potential of AR-based approaches. In the Related Work section (page 2, lines 26–28), we describe SafeSpect in more detail as a prior AR-based interface for drone operation, and clarify how our study differs by focusing on altering the appearance of drones to influence user perceptions and operational behavior.

These additions help readers better understand the background and significance of our research within the context of recent AR-based drone interfaces.

■Regarding Reviewer #2:

Thank you for your comments.
If I summarize the points you raised, I believe they can be outlined as follows.

Q3-1 Novelty, uniqueness, and comparative analysis

A3-1 We appreciate the reviewer’s comment regarding novelty and the need for comparative analysis. The unique contribution of our study is that it employs real drones with AR-modified appearances and quantitatively evaluates both operational behavior and user impressions. While comparative analysis was already described at the end of Section 2 (Related Work), we added a guiding sentence at the beginning of Section 2 to clarify the structure:
“This section first provides an overview of related studies on AR-based appearance modification and drone control interfaces, followed by a discussion of the unique positioning and novelty of the present study.”
This makes it explicit how our study differs from prior work, particularly as no previous studies have dynamically altered the appearance of real drones and analyzed their impact on operational behavior.
For additional details on novelty, please refer to our response to Reviewer #1.

Q3-2 Motivation

A3-2 We thank the reviewer for the suggestion. We agree that the original text mostly presents factual background and could benefit from a more explicit statement of research motivation. To address this, we have added a sentence in the Introduction to clearly highlight our research motivation:
*“Our research motivation is to explore how AR-modified drone appearances can influence user perception and operational behavior, addressing the gap where real drones have not been studied in this context.”*
This addition clarifies why the study was conducted and naturally leads into our research question.

Q3-3 Gender Analysis

A3-4 We conducted an analysis based on participant gender. However, due to page limitations, these results were not explicitly presented in the main manuscript. Participant demographics (12 male, 9 female) are already included in Section 3.5 (Experimental Method), and the gender-based analysis confirmed that there were no significant differences in operational behavior between male and female participants.

Q3-4 Latency Tolerance (Real-time AR Rendering)

A3-4 The goal of our system is to allow users to perceive and operate the AR-projected drones smoothly. To achieve this, we targeted a frame rate of 30 fps (similar to standard video playback), which is sufficient for human operators to perceive motion as continuous and react appropriately. This ensures that AR updates appear visually smooth and minimize perceptual delay during control.

Q3-5 Hardware and AR System Design Considerations

A3-5 We thank the reviewer for these detailed questions. In this study, the AR system operates with real drones in a controlled environment using a single RGB camera and color-based detection (red stickers on the DJI Mini2). No onboard drone sensors or machine learning are used, and GPU acceleration is not applied. AR objects are projected in real time based on detected drone position and controller input, prioritizing low latency and stable tracking. Environmental lighting is controlled via a green screen to maximize detection accuracy. While multi-user viewpoints, predictive object placement, and fully 3D projections are not implemented, they are recognized as important future directions. This simple hardware and software setup effectively demonstrates real-time AR projection for high-speed drones while maintaining operational reliability.

Q3-6 Literature review update

A3-6 We thank the reviewer for the suggestion. We note that a similar comment was also raised by Reviewer #1. In response, we have already added five additional references on recent AR/VR and drone interface research, including SafeSpect (Xu et al., 2025), in the Related Work section. This update clarifies how our study is positioned within the current state-of-the-art.

Q3-7 Use of Machine Learning for AR Appearance

A3-7 We appreciate the reviewer’s insightful questions regarding drift correction, accuracy improvement, and the potential use of machine learning for AR appearance optimization. In our system, we do not employ machine learning or markers; the AR projection is based solely on color detection. This choice was intentional to prioritize processing speed and minimize latency, using a simple and computationally lightweight algorithm. While machine learning could potentially improve accuracy, we consider its integration a future research direction to be explored alongside hardware advancements.

While we have addressed several points such as novelty, motivation, gender analysis, and latency tolerance in the manuscript, the remaining issues regarding detailed psychological evaluation, long-term effects, multi-user scenarios, and advanced technical extensions (e.g., sensor integration, 3D projections, predictive placement) are recognized as important and are positioned as future work.

We have added a sentence addressing this comment at the end of all questionnaire items, on page 14, line 342-350.
