# Supplementary material for: Enhancing user perception and drone flight control through AR-based object projection
Source: PLoS One. 2026 Apr 30;21(4):e0346937. doi: 10.1371/journal.pone.0346937 (PMC13132239; doi:10.1371/journal.pone.0346937)
Supplement: Attachment — Submitted filename: Response_to_Reviewers_auresp_2.docx [file pone.0346937.s003.docx]

Reviewer #3:
Q-6. The manuscript would benefit from a clearer articulation of its novelty relative to prior AR-based drone visualization and acceptance studies in the Introduction and Discussion. Please refer:: Challenges and opportunities on AR/VR technologies for manufacturing systems:: AR/VR assisted integrated framework of autonomous disassembly system.

A-6. Thank you for this helpful suggestion. We reviewed the recommended AR/VR studies and revised the Introduction and Discussion accordingly.

We added citations and clarified that prior AR/VR research mainly focuses on industrial support tasks, static environments, or interface-level augmentation. In contrast, our study investigates real-time appearance modification of physically existing, fast-moving drones and its effects on user perception and operation. These revisions make our novelty clearer.

Q-8. The use of factor analysis is justified; however, the manuscript reports extensive factor loadings and statistical details that may not all be necessary in the main text.

A-8. Thank you for this helpful suggestion. To improve clarity and readability, we have reduced the level of detail presented in the main text and retained only the information necessary to understand the factor structure and interpretation. Detailed factor loadings are now provided in the Supporting Information for transparency and completeness.

Q-9. The limited sample size (n = 21) is acceptable for an exploratory study but should be more clearly framed as such earlier in the manuscript.

A-9. Thank you for this helpful suggestion. We have clarified in the Introduction that this study is positioned as an exploratory study aimed at identifying potential effects and informing future research. This clarification is now provided at the point where the research question and study purpose are introduced.

Q-10. The Results section is comprehensive but overly detailed, with repeated descriptions of statistical outcomes across text, tables, and figures.

A-10. We appreciate this helpful comment. To improve readability and avoid redundancy, we have streamlined the Results section by reducing repeated descriptions of statistical outcomes across the text, tables, and figures. The revised manuscript now focuses on reporting key findings in the main text, while detailed numerical results are primarily presented in tables and figures.

Q-11. Several tables (e.g., detailed factor loadings and effect-size tables) could be moved to Supporting Information to improve readability.

A-11. We appreciate this constructive comment. Following the reviewer’s suggestion, detailed factor loading tables and effect-size tables have been moved to the Supporting Information. The main text now focuses on the key findings, which improves overall readability while still ensuring full transparency through the supplementary materials.

Q-12. Greater emphasis on high-level trends rather than exhaustive numerical reporting would strengthen clarity.

A-12. Thank you for this valuable suggestion. We revised the Results section to emphasize high-level trends rather than detailed numerical reporting. Specifically, we restructured the descriptions so that the main patterns and interpretations are presented first, followed by essential statistical values only where necessary. Redundant numerical details were reduced. These revisions improve clarity and readability while preserving transparency of the analysis.

Q-14. The potential influence of using 2D projections instead of 3D AR could be discussed more explicitly as a limiting factor.

A-14. We thank the reviewer for this insightful comment. We have now explicitly discussed the limitation of using 2D AR projections in the Discussion section. The revised manuscript clarifies that 2D projections provide limited depth cues, which may reduce perceptual realism and partly explain the limited influence on operational behavior. We also note that spatially registered 3D AR is an important direction for future work.

Q-15. The comparison with related work is appropriate, but the implications for real-world drone deployment could be synthesized more concisely.

A-15. We thank the reviewer for this helpful suggestion. We have revised the manuscript to synthesize the implications for real-world drone deployment more concisely. Specifically, we streamlined the discussion of practical applications and focused the Related Work section on positioning the study within prior research. Broader implications for real-world deployment are now summarized more succinctly in the Discussion section.

Q-16. The Conclusion appropriately summarizes the findings but could be shortened by reducing methodological repetition.

A-16. We have revised the Conclusion section to improve conciseness by reducing repetitive descriptions of the experimental methodology. The revised Conclusion now focuses more clearly on the key findings, their implications for drone perception and social acceptance, and future research directions.

Q-17. Future work is well motivated; however, it may be beneficial to more clearly separate near-term extensions (e.g., 3D AR projection) from long-term research directions (e.g., multi-drone scenarios).

A-17. We reorganized the Future Work section by clearly distinguishing near-term extensions, such as validation with controlled experiments and the use of 3D AR projections, from longer-term research directions, including multi-drone scenarios, long-term exposure, and real-world deployment. This restructuring improves clarity and better reflects the intended research roadmap.

Q-18. The manuscript is generally well written, though minor grammatical errors and formatting inconsistencies are present.

Q-18. we conducted a full read-through to correct minor grammatical errors and formatting inconsistencies throughout the manuscript.

Q-19. Repetition of certain sentences and phrases should be reduced, particularly in the Introduction and Results sections.

A- 19. we reduced unnecessary repetition, particularly in the Introduction and Results sections, while retaining intentional emphasis where it contributes to clarity and narrative flow.

Q-20. Terminology should be reviewed for consistency (e.g., “appearance modification,” “object projection,” “AR projection”).

Thank you for this suggestion. We have revised the manuscript to use **“AR-based appearance modification”** consistently throughout the text. This unifies the terminology and clarifies that all references to drone appearance changes refer to modifications applied via AR.
